# *catena*-Poly[[4′,5′-bis­(methyl­sulfan­yl)-4,5-ethyl­enedithio­tetra­thio­fulvalene] [[dichloridomercurate(II)]-μ-dichlorido]]

**DOI:** 10.1107/S1600536808042785

**Published:** 2008-12-20

**Authors:** Wei Yang, Zhi-Gang Ren, Jie Dai

**Affiliations:** a Department of Basic Course, Suzhou Polytechnical Institute of Agriculture, Suzhou 215008, People’s Republic of China; b College of Chemistry, Chemical Engineering and Materials Science, Soochow University, Suzhou 215123, People’s Republic of China

## Abstract

The title compound, {(C_10_H_10_S_8_)[HgCl_3_]}_*n*_, is a sulfur-rich charge-transfer compound in which C_10_H_10_S_8_^+^ cations and HgCl_3_ anions are assembled by S⋯S [3.371 (5)–3.588 (5) Å] and S⋯Cl [2.833 (4)–3.488 (4) Å] contacts, and by weak inter­molecular C—H⋯Cl hydrogen bonds, forming a two-dimensional wave-like structure. The two C atoms of the –CH_2_—CH_2_– group in one of the cations are disordered over two sites with relative occupancies of 0.83 (2) and 0.17 (2).

## Related literature

For background information, see: Banks *et al.* (1978 ▶); Enomoto *et al.* (2001 ▶); Kistenmacher *et al*. (1980 ▶); Zhilyaeva *et al.* (1999 ▶). For realted structures, see: Zhang *et al.* (1996 ▶); Hudhomme *et al.* (2001 ▶); Wu *et al.* (1998 ▶); Aakeröy *et al.* (1999 ▶).
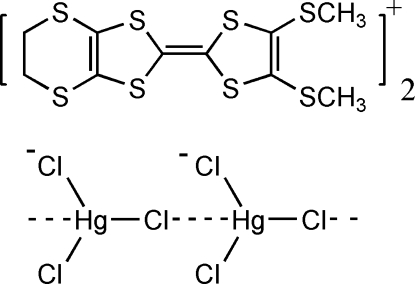


## Experimental

### 

#### Crystal data

(C_10_H_10_S_8_)[HgCl_3_]
*M* *_r_* = 693.6
Monoclinic, 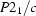

*a* = 7.7216 (15) Å
*b* = 25.541 (5) Å
*c* = 19.626 (4) Å
β = 97.96 (3)°
*V* = 3833.3 (13) Å^3^
*Z* = 8
Mo *K*α radiation
μ = 9.31 mm^−1^
*T* = 193 (2) K
0.30 × 0.06 × 0.05 mm

#### Data collection

Rigaku Mercury CCD diffractometer
Absorption correction: multi-scan (Jacobson, 1998 ▶) *T*_min_ = 0.167, *T*_max_ = 0.653
34949 measured reflections
6730 independent reflections
6416 reflections with *I* > 2σ(*I*)
*R*_int_ = 0.081

#### Refinement

*R*[*F* ^2^ > 2σ(*F* ^2^)] = 0.097
*wR*(*F* ^2^) = 0.158
*S* = 1.70
6730 reflections
393 parameters
7 restraints
H-atom parameters constrained
Δρ_max_ = 1.24 e Å^−3^
Δρ_min_ = −1.37 e Å^−3^

### 

Data collection: *CrystalClear* (Rigaku, 2001 ▶); cell refinement: *CrystalClear*; data reduction: *CrystalClear*; program(s) used to solve structure: *SHELXS97* (Sheldrick, 2008 ▶); program(s) used to refine structure: *SHELXL97* (Sheldrick, 2008 ▶); molecular graphics: *ORTEPII* (Johnson, 1976 ▶); software used to prepare material for publication: *SHELXL97*.

## Supplementary Material

Crystal structure: contains datablocks I, global. DOI: 10.1107/S1600536808042785/lh2724sup1.cif

Structure factors: contains datablocks I. DOI: 10.1107/S1600536808042785/lh2724Isup2.hkl

Additional supplementary materials:  crystallographic information; 3D view; checkCIF report

## Figures and Tables

**Table 1:** Selected bond lengths (Å)

Hg1—Cl1	2.384 (4)
Hg1—Cl2	2.411 (4)
Hg1—Cl3	2.516 (4)
Hg2—Cl3	2.833 (4)
Hg2—Cl4	2.409 (4)
Hg2—Cl5	2.403 (4)
Hg2—Cl6	2.483 (4)

**Table 2:** Hydrogen-bond geometry (Å, °)

*D*—H⋯*A*	*D*—H	H⋯*A*	*D*⋯*A*	*D*—H⋯*A*
C2—H2*A*⋯Cl4^i^	0.99	2.76	3.619 (19)	146
C2—H2*B*⋯Cl3^ii^	0.99	2.68	3.645 (19)	164

Symmetry codes: (i) 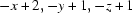; (ii) 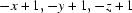.

**Fig. 1.**
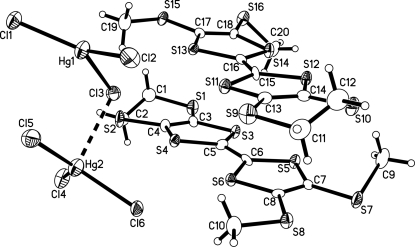
The molecular structure of (I), with the atom-numbering scheme. Displacement ellipsoids are drawn at the 50% probability level and H atoms are shown as small spheres of arbitrary radii. The disordered C and H atoms are omitted.

**Fig. 2.**
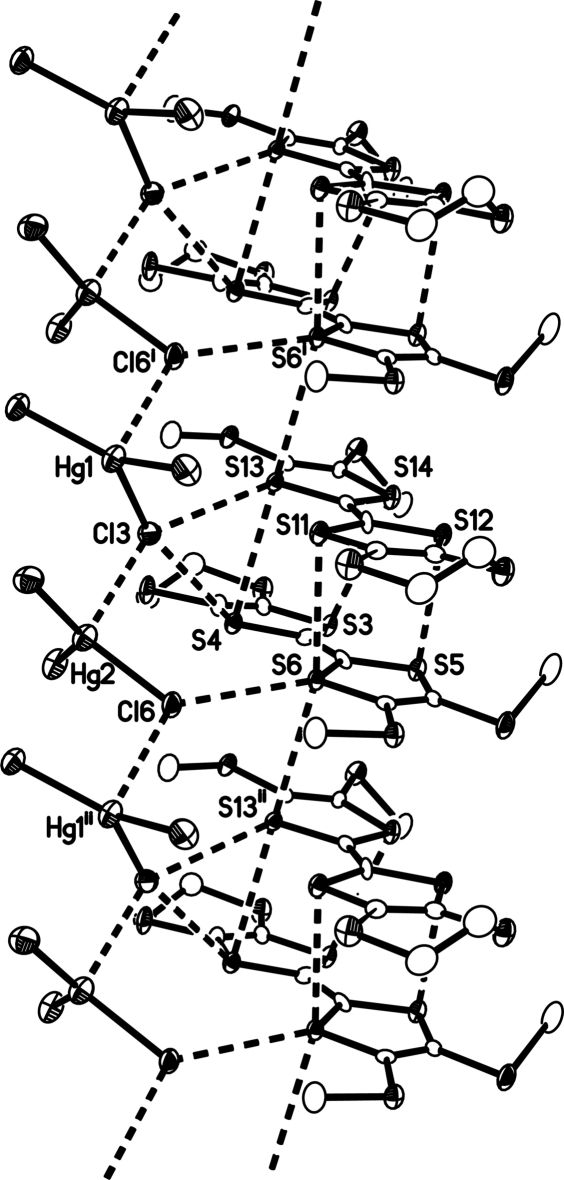
Part of the crystal structure showing the face-to-face stacking of DMTEDT-TTF+ cations (connected by S···S interactions) and the one-dimensional chain of the HgCl3- anions (connected by Hg···Cl interactions) along a axis. The stacking and the chain are linked by intramolecular S···Cl interactions. All H atoms have been omitted and the disorder is not shown. [symmetry codes: (i)x - 1,y,z; (ii)x + 1,y,z]

**Fig. 3.**
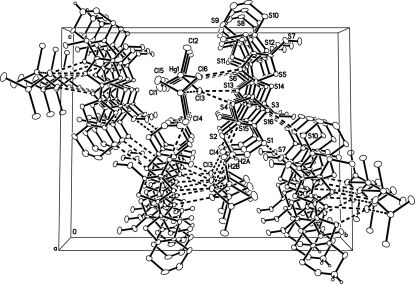
Part of the crystal structure of (I). The intermolecular S···S, S···Cl interactions (dashed lines) and H-bonds connect the DMTEDT-TTF+ stacks and the HgCl3- chains into a two-dimensional 'wave-like' structure. All non-hydrogen bonding H atoms have been omitted. The disorder is not showm.

## supplementary crystallographic information

### Comment

Although tetrathiafulvalene (TTF) and its radical salts have been investigated for several decades, they are still attracting much attention from chemists. The TTF unit can exist in three stable redox-states (TTF / TTF^+^/ TTF^2+^) and for this reason their derivatives have been used as functional building blocks in supramolecular chemistry and materials chemistry. There are two synthetic routes to prepare the oxidized TTF derivatives: chemical oxidization and electrochemical oxidization. It is known that HgCl_2_ can oxidize the TTF derivatives readily, forming a set of charge-transfer (CT) salts (Banks *et al.* 1978; Enomoto *et al.* 2001; Kistenmacher *et al.* 1980; Zhilyaeva *et al.* 1999). The chloromercurate anions are found to have monomeric, dimeric or polymeric structures. In this paper, we report the synthesis and crystal structure of a new charge-transfer salt (I).

Compound (I) consists of two DMTEDT-TTF^+^ cations and two HgCl_3_^-^ anions (Fig. 1). Unlike the precursor DMTEDT-TTF (Zhang *et al.*, 1996), each DMTEDT-TTF^+^ cation is nearly co-planar through the conjugated TTF moiety (bis(dithio)tetrathiofulvalene, C_6_S_8_) with the maximum deviation of 0.152 (4) Å (S1) and 0.179 (4) Å (S10). Compared with those of the molecule DMTEDT-TTF, the bond lengths of the conjugated systems in (I) are averaged which are close to those of DMTEDT-TTF^+^ perchlorate (Hudhomme *et al.*, 2001). The central C═C bond distance of the TTF unit is the charge-sensitive parameter for the electronic states of the TTF derivatives. The distances were reported to be 1.33–1.35 Å for TTCn-TTF^0^, 1.38–1.40 Å for TTCn-TTF^+^ and 1.42–1.43 Å for TTCn-TTF^2+^, respectively (Wu *et al.*, 1998). The C═C distances in (I) are 1.403 (18) Å (C5═C6) and 1.390 (17) Å (C15═C16), which correspond to the monovalent cation. The two cations are almost parallel but oriented in opposite direction. The dihedral angle between the least-squires planes of TTF moieties is 1.77 (7)°. Four strong intramolecular S···S contacts (S3···S14 3.508 (5) Å; S4···S13 3.382 (5) Å; S5···S12 3.513 (5) Å; S6···S11 3.371 (5) Å) and an intermolecular S···S interaction (S13···S6^i^ 3.532 (5) Å) connect the cations into a one-dimensional chain extending along the *a* axis (Fig. 2). The HgCl_3_ anions are linked *via* the intramolecular Hg2···Cl3 (2.833 (4) Å) and intermolecular Hg1···Cl6^i^ (2.901 (4) Å) secondary bonding interactions, thereby forming a one-dimensional [HgCl_3_^-^]_n_ chain extending along the *a* axis. The DMTEDT-TTF moiety interacts with the one-dimensional chain by three intramolecular S···Cl interactions (S4···Cl3 3.440 (5) Å; S6···Cl6 3.435 (5) Å; S13···Cl3 3.488 (5) Å, Fig. 2). It seems like that the [HgCl_3_^-^]_n_ chain is stalibized by two rings: a 8-member Hg2—Cl6—S6—C6—C5—S4—Cl3 ring and a 5-member Hg1—Cl3—S13—S6^i^—Cl6^i^ ring, which are linked alternatively by the S···S contacts mentioned above. Between the stacking and the chain there are several side-to-side intermolecular interactions (S15···Cl4^iv^ 3.425 (5) Å; S1···S7^v^ 3.438 (5) Å; S3···S10^vi^ 3.588 (5) Å) and two intermolecular C—H···Cl hydrogen bonding interactions (Aakeröy *et al.* 1999) which result in the formation of a wave-like two-dimensional structure as shown in Fig. 3 [symmetry codes: (i)*x* - 1,*y*,*z*; (iv)-*x* + 1,-*y* + 1,-*z* + 1; (v)*x* - 1,-*y* + 3/2,*z* - 1/2; (vi)*x*,-*y* + 3/2,*z* - 1/2].

### Experimental

A solution of HgCl_2_ (5.7 mg, 0.02 mmol) in MeCN (2 ml) was added into the solution of DMTEDT-TTF (bis(methylthio)ethylenedithiotetrathiafulvalene, C_10_H_10_H_8_, (4.0 mg, 0.01 mmol) in CH_2_Cl_2_ (2 ml). Slow evaporation of solevnts from the resulting orange solution for 3 days afforded dark blue prisms of (I). Yield: 4.9 mg (71%). CH&N elemental analysis. Found: C, 17.02; H, 1.64. Calculated for C_20_H_20_Cl_6_Hg_2_S_16_: C, 17.31; H, 1.45%.

### Refinement

Two carbon atoms of one DMTEDT-TTF group are disordered over two orientations with occupancy factors of 0.83/0.17 for C1/C1A and C2/C2A. These two disordered C atoms are refined isotropically, while all other non-hydrogen atoms are refined anisotropically. The H atoms are placed in geometrically idealized positions (C—H = 0.98 Å for methyl groups and C—H = 0.99 Å for methylene groups) and constrained to ride on their parent atoms with *U*_iso_(H) = 1.2*U*_eq_(C).

### Crystal data

**Table:**

(C_10_H_10_S_8_)[HgCl_3_]	*F*(000) = 2632
*M**_r_* = 693.6	*D*_x_ = 2.404 Mg m^−^^3^
Monoclinic, *P*2_1_/*c*	Mo *K*α radiation, λ = 0.71073 Å
Hall symbol: -P 2ybc	Cell parameters from 5236 reflections
*a* = 7.7216 (15) Å	θ = 3.1–25.0°
*b* = 25.541 (5) Å	µ = 9.31 mm^−^^1^
*c* = 19.626 (4) Å	*T* = 193 K
β = 97.96 (3)°	Prism, blue
*V* = 3833.3 (13) Å^3^	0.30 × 0.06 × 0.05 mm
*Z* = 8	

### Data collection

**Table:**

Rigaku Mercury CCD diffractometer	6730 independent reflections
Radiation source: fine-focus sealed tube	6416 reflections with *I* > 2σ(*I*)
graphite	*R*_int_ = 0.081
ω scans	θ_max_ = 25.0°, θ_min_ = 3.1°
Absorption correction: multi-scan (Jacobson, 1998)	*h* = −9→9
*T*_min_ = 0.167, *T*_max_ = 0.653	*k* = −30→30
34949 measured reflections	*l* = −23→23

### Refinement

**Table:**

Refinement on *F*^2^	Primary atom site location: structure-invariant direct methods
Least-squares matrix: full	Secondary atom site location: difference Fourier map
*R*[*F*^2^ > 2σ(*F*^2^)] = 0.097	Hydrogen site location: inferred from neighbouring sites
*wR*(*F*^2^) = 0.158	H-atom parameters constrained
*S* = 1.70	*w* = 1/[σ^2^(*F*_o_^2^) + (0.041*P*)^2^ + 19.9*P*] where *P* = (*F*_o_^2^ + 2*F*_c_^2^)/3
6730 reflections	(Δ/σ)_max_ = 0.001
393 parameters	Δρ_max_ = 1.24 e Å^−^^3^
7 restraints	Δρ_min_ = −1.37 e Å^−^^3^

### Special details

**Table:**

Geometry. All e.s.d.'s (except the e.s.d. in the dihedral angle between two l.s. planes) are estimated using the full covariance matrix. The cell e.s.d.'s are taken into account individually in the estimation of e.s.d.'s in distances, angles and torsion angles; correlations between e.s.d.'s in cell parameters are only used when they are defined by crystal symmetry. An approximate (isotropic) treatment of cell e.s.d.'s is used for estimating e.s.d.'s involving l.s. planes.
Refinement. Refinement of *F*^2^ against ALL reflections. The weighted *R*-factor *wR* and goodness of fit *S* are based on *F*^2^, conventional *R*-factors *R* are based on *F*, with *F* set to zero for negative *F*^2^. The threshold expression of *F*^2^ > σ(*F*^2^) is used only for calculating *R*-factors(gt) *etc*. and is not relevant to the choice of reflections for refinement. *R*-factors based on *F*^2^ are statistically about twice as large as those based on *F*, and *R*- factors based on ALL data will be even larger.

### Fractional atomic coordinates and isotropic or equivalent isotropic displacement parameters (Å^2^)

**Table:**

	*x*	*y*	*z*	*U*_iso_*/*U*_eq_	Occ. (<1)
Hg1	0.45533 (8)	0.41687 (2)	0.78044 (3)	0.02899 (18)	
Hg2	0.91933 (8)	0.42035 (2)	0.70386 (3)	0.02898 (18)	
Cl1	0.2922 (5)	0.34433 (16)	0.7290 (2)	0.0399 (10)	
Cl2	0.5596 (5)	0.43869 (17)	0.89856 (19)	0.0373 (10)	
Cl3	0.6117 (4)	0.47942 (13)	0.71089 (19)	0.0267 (8)	
Cl4	0.9288 (5)	0.43697 (15)	0.58359 (19)	0.0344 (9)	
Cl5	0.7983 (5)	0.34659 (16)	0.7568 (2)	0.0384 (10)	
Cl6	1.1374 (5)	0.47851 (14)	0.77105 (19)	0.0289 (8)	
S1	0.6081 (5)	0.68944 (14)	0.48929 (18)	0.0254 (8)	
S2	0.6520 (5)	0.55351 (14)	0.52101 (19)	0.0281 (9)	
S3	0.7971 (4)	0.69856 (13)	0.63019 (16)	0.0177 (7)	
S4	0.8238 (4)	0.58695 (13)	0.65687 (17)	0.0181 (7)	
S5	1.0073 (4)	0.72162 (13)	0.78194 (17)	0.0195 (7)	
S6	1.0639 (4)	0.60862 (13)	0.80084 (16)	0.0158 (7)	
S7	1.2347 (5)	0.74770 (15)	0.91583 (19)	0.0291 (9)	
S8	1.2877 (5)	0.62303 (13)	0.93929 (17)	0.0208 (8)	
S9	0.8532 (5)	0.55476 (15)	0.99144 (19)	0.0302 (9)	
S10	0.8668 (5)	0.69012 (15)	1.02585 (18)	0.0272 (8)	
S11	0.6767 (4)	0.58628 (13)	0.85657 (17)	0.0194 (7)	
S12	0.6762 (4)	0.69875 (13)	0.88435 (16)	0.0184 (7)	
S13	0.4332 (4)	0.60475 (12)	0.71297 (16)	0.0155 (7)	
S14	0.4648 (4)	0.71822 (13)	0.73263 (17)	0.0191 (7)	
S15	0.2034 (4)	0.61569 (13)	0.57563 (17)	0.0200 (7)	
S16	0.2282 (5)	0.74147 (14)	0.60020 (18)	0.0247 (8)	
C3	0.7079 (17)	0.6574 (5)	0.5647 (7)	0.018 (3)	
C4	0.7214 (17)	0.6054 (5)	0.5764 (7)	0.019 (3)	
C5	0.8706 (16)	0.6496 (5)	0.6860 (7)	0.016 (3)	
C6	0.9700 (16)	0.6589 (5)	0.7503 (7)	0.017 (3)	
C7	1.1340 (16)	0.7023 (5)	0.8570 (6)	0.013 (3)	
C8	1.1591 (15)	0.6493 (5)	0.8665 (6)	0.014 (3)	
C9	1.067 (2)	0.7954 (6)	0.9159 (8)	0.034 (4)	
H9A	1.1078	0.8237	0.9478	0.051*	
H9B	0.9630	0.7790	0.9305	0.051*	
H9C	1.0366	0.8097	0.8695	0.051*	
C10	1.281 (2)	0.5544 (6)	0.9207 (7)	0.028 (3)	
H10A	1.3497	0.5353	0.9585	0.042*	
H10B	1.3296	0.5480	0.8779	0.042*	
H10C	1.1593	0.5422	0.9154	0.042*	
C11	0.957 (2)	0.5888 (6)	1.0678 (8)	0.036 (4)	
H11A	0.9676	0.5643	1.1073	0.043*	
H11B	1.0765	0.5992	1.0606	0.043*	
C12	0.859 (2)	0.6364 (7)	1.0853 (7)	0.036 (4)	
H12A	0.9084	0.6484	1.1320	0.043*	
H12B	0.7355	0.6268	1.0864	0.043*	
C13	0.7717 (16)	0.6061 (5)	0.9379 (6)	0.015 (3)	
C14	0.7783 (16)	0.6589 (5)	0.9497 (7)	0.017 (3)	
C15	0.6144 (17)	0.6478 (5)	0.8274 (6)	0.016 (3)	
C16	0.5164 (16)	0.6568 (5)	0.7635 (6)	0.014 (3)	
C17	0.3223 (16)	0.6446 (5)	0.6483 (6)	0.015 (3)	
C18	0.3376 (16)	0.6969 (5)	0.6581 (6)	0.014 (3)	
C19	0.228 (2)	0.5470 (5)	0.5954 (7)	0.027 (3)	
H19A	0.1648	0.5264	0.5576	0.041*	
H19B	0.1795	0.5395	0.6380	0.041*	
H19C	0.3520	0.5376	0.6013	0.041*	
C20	0.3862 (19)	0.7930 (5)	0.5994 (7)	0.025 (3)	
H20A	0.3375	0.8208	0.5679	0.037*	
H20B	0.4923	0.7790	0.5839	0.037*	
H20C	0.4151	0.8074	0.6458	0.037*	
C1	0.508 (2)	0.6359 (6)	0.4384 (9)	0.030 (3)*	0.83 (2)
H1A	0.3974	0.6265	0.4556	0.036*	0.83 (2)
H1B	0.4781	0.6479	0.3902	0.036*	0.83 (2)
C2	0.621 (3)	0.5870 (7)	0.4389 (8)	0.030 (3)*	0.83 (2)
H2A	0.7364	0.5969	0.4267	0.036*	0.83 (2)
H2B	0.5655	0.5625	0.4032	0.036*	0.83 (2)
C1A	0.612 (12)	0.6349 (19)	0.431 (3)	0.030 (3)*	0.17 (2)
H1C	0.5589	0.6458	0.3846	0.036*	0.17 (2)
H1D	0.7356	0.6253	0.4287	0.036*	0.17 (2)
C2A	0.516 (8)	0.587 (3)	0.453 (3)	0.030 (3)*	0.17 (2)
H2D	0.4882	0.5632	0.4127	0.036*	0.17 (2)
H2C	0.4052	0.5978	0.4682	0.036*	0.17 (2)

### Atomic displacement parameters (Å^2^)

**Table:**

	*U*^11^	*U*^22^	*U*^33^	*U*^12^	*U*^13^	*U*^23^
Hg1	0.0336 (3)	0.0270 (3)	0.0250 (3)	−0.0062 (3)	−0.0007 (2)	−0.0006 (3)
Hg2	0.0341 (4)	0.0281 (3)	0.0241 (3)	−0.0064 (3)	0.0021 (2)	0.0027 (3)
Cl1	0.029 (2)	0.031 (2)	0.056 (3)	−0.0062 (17)	−0.0026 (18)	−0.0127 (19)
Cl2	0.036 (2)	0.047 (3)	0.025 (2)	0.0104 (19)	−0.0085 (16)	−0.0051 (17)
Cl3	0.0252 (18)	0.0184 (18)	0.037 (2)	0.0013 (14)	0.0049 (15)	0.0038 (15)
Cl4	0.048 (2)	0.031 (2)	0.0234 (19)	−0.0109 (18)	0.0014 (17)	0.0027 (16)
Cl5	0.036 (2)	0.029 (2)	0.052 (3)	−0.0013 (17)	0.0115 (19)	0.0120 (19)
Cl6	0.0265 (19)	0.0238 (19)	0.033 (2)	0.0029 (15)	−0.0086 (15)	−0.0099 (15)
S1	0.033 (2)	0.021 (2)	0.0196 (18)	0.0012 (16)	−0.0071 (15)	0.0011 (15)
S2	0.037 (2)	0.018 (2)	0.025 (2)	−0.0005 (16)	−0.0087 (16)	−0.0092 (15)
S3	0.0232 (18)	0.0132 (17)	0.0151 (16)	−0.0030 (14)	−0.0029 (13)	0.0003 (13)
S4	0.0211 (17)	0.0111 (17)	0.0208 (17)	0.0000 (14)	−0.0022 (13)	0.0019 (13)
S5	0.0237 (18)	0.0144 (17)	0.0184 (17)	−0.0015 (14)	−0.0036 (14)	0.0002 (13)
S6	0.0192 (17)	0.0118 (16)	0.0153 (16)	0.0015 (13)	−0.0011 (13)	0.0003 (13)
S7	0.030 (2)	0.024 (2)	0.029 (2)	0.0002 (16)	−0.0115 (16)	−0.0100 (16)
S8	0.0262 (19)	0.0195 (19)	0.0144 (17)	0.0020 (15)	−0.0055 (14)	−0.0003 (14)
S9	0.039 (2)	0.022 (2)	0.027 (2)	0.0066 (17)	−0.0032 (17)	0.0106 (16)
S10	0.032 (2)	0.027 (2)	0.0189 (18)	−0.0011 (16)	−0.0076 (15)	−0.0023 (15)
S11	0.0235 (18)	0.0139 (17)	0.0195 (17)	−0.0004 (14)	−0.0016 (14)	0.0016 (14)
S12	0.0226 (18)	0.0158 (18)	0.0148 (17)	−0.0006 (14)	−0.0046 (14)	−0.0013 (13)
S13	0.0158 (16)	0.0126 (16)	0.0171 (17)	−0.0002 (13)	−0.0013 (13)	0.0006 (13)
S14	0.0260 (19)	0.0138 (17)	0.0148 (17)	0.0010 (14)	−0.0063 (14)	−0.0001 (13)
S15	0.0243 (18)	0.0177 (18)	0.0164 (17)	−0.0046 (14)	−0.0032 (14)	0.0005 (13)
S16	0.0281 (19)	0.0177 (19)	0.0250 (19)	−0.0005 (15)	−0.0077 (15)	0.0049 (15)
C3	0.019 (7)	0.018 (7)	0.016 (7)	0.000 (6)	0.004 (5)	−0.006 (6)
C4	0.020 (7)	0.018 (7)	0.015 (7)	0.004 (6)	−0.009 (5)	−0.004 (6)
C5	0.015 (7)	0.013 (7)	0.021 (7)	−0.002 (5)	0.004 (5)	0.007 (5)
C6	0.019 (7)	0.012 (7)	0.022 (7)	0.007 (5)	0.006 (6)	0.000 (6)
C7	0.013 (6)	0.020 (7)	0.008 (6)	0.002 (5)	0.001 (5)	−0.004 (5)
C8	0.009 (6)	0.026 (8)	0.007 (6)	0.003 (5)	−0.001 (5)	0.000 (5)
C9	0.045 (10)	0.013 (8)	0.039 (9)	−0.006 (7)	−0.008 (7)	0.001 (7)
C10	0.041 (9)	0.022 (8)	0.020 (8)	0.003 (7)	−0.004 (7)	0.003 (6)
C11	0.034 (9)	0.035 (10)	0.034 (9)	0.005 (7)	−0.015 (7)	0.010 (7)
C12	0.041 (10)	0.051 (11)	0.014 (8)	0.000 (8)	−0.004 (7)	0.008 (7)
C13	0.016 (7)	0.020 (7)	0.010 (6)	0.000 (6)	0.004 (5)	0.005 (5)
C14	0.012 (7)	0.020 (7)	0.018 (7)	0.005 (5)	−0.002 (5)	0.002 (6)
C15	0.022 (7)	0.010 (7)	0.016 (7)	0.005 (5)	−0.003 (5)	−0.005 (5)
C16	0.015 (7)	0.012 (7)	0.017 (7)	0.002 (5)	0.007 (5)	0.002 (5)
C17	0.013 (6)	0.021 (7)	0.010 (6)	0.003 (5)	−0.001 (5)	−0.004 (5)
C18	0.013 (6)	0.014 (7)	0.014 (7)	−0.001 (5)	−0.001 (5)	−0.004 (5)
C19	0.038 (9)	0.017 (8)	0.025 (8)	−0.001 (6)	0.001 (7)	0.005 (6)
C20	0.037 (9)	0.013 (7)	0.023 (8)	−0.010 (6)	0.000 (6)	0.005 (6)

### Geometric parameters (Å, °)

**Table:**

Hg1—Cl1	2.384 (4)	S14—C18	1.733 (12)
Hg1—Cl2	2.411 (4)	S15—C17	1.749 (12)
Hg1—Cl3	2.516 (4)	S15—C19	1.801 (14)
Hg1—Cl6^i^	2.901 (4)	S16—C18	1.743 (13)
Hg2—Cl3	2.833 (4)	S16—C20	1.797 (14)
Hg2—Cl4	2.409 (4)	C3—C4	1.348 (19)
Hg2—Cl5	2.403 (4)	C5—C6	1.403 (18)
Hg2—Cl6	2.483 (4)	C7—C8	1.375 (18)
Cl6—Hg1^ii^	2.901 (4)	C9—H9A	0.9800
S1—C3	1.772 (14)	C9—H9B	0.9800
S1—C1A	1.80 (2)	C9—H9C	0.9800
S1—C1	1.804 (15)	C10—H10A	0.9800
S2—C4	1.750 (13)	C10—H10B	0.9800
S2—C2A	1.80 (2)	C10—H10C	0.9800
S2—C2	1.810 (14)	C11—C12	1.50 (2)
S3—C5	1.707 (13)	C11—H11A	0.9900
S3—C3	1.728 (13)	C11—H11B	0.9900
S4—C4	1.731 (13)	C12—H12A	0.9900
S4—C5	1.720 (13)	C12—H12B	0.9900
S5—C6	1.728 (13)	C13—C14	1.367 (18)
S5—C7	1.724 (12)	C15—C16	1.390 (17)
S6—C6	1.720 (13)	C17—C18	1.353 (18)
S6—C8	1.738 (13)	C19—H19A	0.9800
S7—C7	1.741 (13)	C19—H19B	0.9800
S7—C9	1.780 (16)	C19—H19C	0.9800
S8—C8	1.757 (12)	C20—H20A	0.9800
S8—C10	1.791 (15)	C20—H20B	0.9800
S9—C13	1.744 (13)	C20—H20C	0.9800
S9—C11	1.820 (16)	C1—C2	1.52 (2)
S10—C14	1.747 (13)	C1—H1A	0.9900
S10—C12	1.806 (16)	C1—H1B	0.9900
S11—C13	1.737 (13)	C2—H2A	0.9900
S11—C15	1.717 (13)	C2—H2B	0.9900
S12—C14	1.739 (13)	C1A—C2A	1.52 (3)
S12—C15	1.739 (13)	C1A—H1C	0.9900
S13—C16	1.728 (13)	C1A—H1D	0.9900
S13—C17	1.755 (13)	C2A—H2D	0.9900
S14—C16	1.709 (13)	C2A—H2C	0.9900
			
Cl1—Hg1—Cl2	132.27 (16)	C12—C11—H11A	108.9
Cl1—Hg1—Cl3	121.93 (14)	S9—C11—H11A	108.9
Cl2—Hg1—Cl3	104.68 (14)	C12—C11—H11B	108.9
Cl1—Hg1—Cl6^i^	90.12 (12)	S9—C11—H11B	108.9
Cl2—Hg1—Cl6^i^	95.88 (12)	H11A—C11—H11B	107.7
Cl3—Hg1—Cl6^i^	94.96 (11)	C11—C12—S10	114.0 (11)
Cl5—Hg2—Cl4	128.94 (14)	C11—C12—H12A	108.7
Cl5—Hg2—Cl6	121.00 (14)	S10—C12—H12A	108.7
Cl4—Hg2—Cl6	107.73 (13)	C11—C12—H12B	108.7
Cl5—Hg2—Cl3	91.01 (12)	S10—C12—H12B	108.7
Cl4—Hg2—Cl3	95.35 (13)	H12A—C12—H12B	107.6
Cl6—Hg2—Cl3	99.61 (11)	C14—C13—S11	116.4 (10)
Hg1—Cl3—Hg2	99.03 (11)	C14—C13—S9	129.6 (10)
Hg2—Cl6—Hg1^ii^	102.40 (12)	S11—C13—S9	113.9 (8)
C3—S1—C1A	97 (3)	C13—C14—S12	116.7 (10)
C3—S1—C1	102.4 (8)	C13—C14—S10	126.6 (10)
C1A—S1—C1	27 (3)	S12—C14—S10	116.6 (8)
C4—S2—C2A	102 (3)	C16—C15—S11	123.0 (10)
C4—S2—C2	100.7 (8)	C16—C15—S12	121.5 (10)
C2A—S2—C2	28 (2)	S11—C15—S12	115.5 (7)
C5—S3—C3	95.4 (7)	C15—C16—S14	122.9 (10)
C5—S4—C4	95.7 (6)	C15—C16—S13	120.2 (10)
C7—S5—C6	95.1 (6)	S14—C16—S13	116.9 (7)
C6—S6—C8	94.7 (6)	C18—C17—S15	124.0 (10)
C7—S7—C9	101.4 (7)	C18—C17—S13	116.4 (9)
C8—S8—C10	102.2 (6)	S15—C17—S13	119.6 (8)
C13—S9—C11	102.6 (7)	C17—C18—S14	117.3 (10)
C14—S10—C12	99.3 (7)	C17—C18—S16	121.8 (10)
C15—S11—C13	95.9 (6)	S14—C18—S16	120.8 (7)
C14—S12—C15	95.3 (6)	S15—C19—H19A	109.5
C16—S13—C17	94.3 (6)	S15—C19—H19B	109.5
C16—S14—C18	95.0 (6)	H19A—C19—H19B	109.5
C17—S15—C19	102.0 (7)	S15—C19—H19C	109.5
C18—S16—C20	102.5 (6)	H19A—C19—H19C	109.5
C4—C3—S3	117.3 (10)	H19B—C19—H19C	109.5
C4—C3—S1	127.7 (10)	S16—C20—H20A	109.5
S3—C3—S1	115.0 (8)	S16—C20—H20B	109.5
C3—C4—S4	116.0 (10)	H20A—C20—H20B	109.5
C3—C4—S2	129.1 (10)	S16—C20—H20C	109.5
S4—C4—S2	115.0 (8)	H20A—C20—H20C	109.5
C6—C5—S3	123.0 (10)	H20B—C20—H20C	109.5
C6—C5—S4	121.3 (10)	C2—C1—S1	114.7 (12)
S3—C5—S4	115.6 (8)	C2—C1—H1A	108.6
C5—C6—S6	121.8 (10)	S1—C1—H1A	108.6
C5—C6—S5	121.6 (10)	C2—C1—H1B	108.6
S6—C6—S5	116.7 (8)	S1—C1—H1B	108.6
C8—C7—S5	116.7 (9)	H1A—C1—H1B	107.6
C8—C7—S7	121.6 (9)	C1—C2—S2	113.6 (12)
S5—C7—S7	121.6 (8)	C1—C2—H2A	108.8
C7—C8—S6	116.8 (9)	S2—C2—H2A	108.8
C7—C8—S8	122.6 (9)	C1—C2—H2B	108.8
S6—C8—S8	120.5 (8)	S2—C2—H2B	108.8
S7—C9—H9A	109.5	H2A—C2—H2B	107.7
S7—C9—H9B	109.5	C2A—C1A—S1	113 (5)
H9A—C9—H9B	109.5	C2A—C1A—H1C	108.9
S7—C9—H9C	109.5	S1—C1A—H1C	108.9
H9A—C9—H9C	109.5	C2A—C1A—H1D	108.9
H9B—C9—H9C	109.5	S1—C1A—H1D	108.9
S8—C10—H10A	109.5	H1C—C1A—H1D	107.7
S8—C10—H10B	109.5	C1A—C2A—S2	109 (5)
H10A—C10—H10B	109.5	C1A—C2A—H2D	109.8
S8—C10—H10C	109.5	S2—C2A—H2D	109.8
H10A—C10—H10C	109.5	C1A—C2A—H2C	109.8
H10B—C10—H10C	109.5	S2—C2A—H2C	109.8
C12—C11—S9	113.5 (10)	H2D—C2A—H2C	108.2

Symmetry codes: (i) *x*−1, *y*, *z*; (ii) *x*+1, *y*, *z*.

### Hydrogen-bond geometry (Å, °)

**Table:**

*D*—H···*A*	*D*—H	H···*A*	*D*···*A*	*D*—H···*A*
C2—H2A···Cl4^iii^	0.99	2.76	3.619 (19)	146
C2—H2B···Cl3^iv^	0.99	2.68	3.645 (19)	164

Symmetry codes: (iii) −*x*+2, −*y*+1, −*z*+1; (iv) −*x*+1, −*y*+1, −*z*+1.
